# Mechanistic insights into SGLT2 inhibition in Takotsubo syndrome

**DOI:** 10.1093/ehjcvp/pvag017

**Published:** 2026-03-25

**Authors:** Antonin Trimaille, Taraneh Tatarcheh, Amandine Granier, Valérie Schini-Kerth, Olivier Morel

**Affiliations:** Department of Cardiovascular Medicine, Nouvel Hôpital Civil, Strasbourg University Hospital, 1 place de l’Hôpital, Strasbourg 67000, France; UR 3074 Translational Cardiovascular Medicine, CRBS, University of Strasbourg, Strasbourg 67000, France; UR 3074 Translational Cardiovascular Medicine, CRBS, University of Strasbourg, Strasbourg 67000, France; Department of Cardiovascular Medicine, Nouvel Hôpital Civil, Strasbourg University Hospital, 1 place de l’Hôpital, Strasbourg 67000, France; UR 3074 Translational Cardiovascular Medicine, CRBS, University of Strasbourg, Strasbourg 67000, France; Department of Cardiovascular Medicine, Nouvel Hôpital Civil, Strasbourg University Hospital, 1 place de l’Hôpital, Strasbourg 67000, France; UR 3074 Translational Cardiovascular Medicine, CRBS, University of Strasbourg, Strasbourg 67000, France

**This correspondence refers to ‘SGLT2 inhibitors are associated with improved long-term survival in Takotsubo syndrome: insights from large-scale real-world data’, by E. Rawish**  ***et al.,*****https://doi.org/10.1093/ehjcvp/pvaf088.**

To the Editor:

We read with great interest the recent article by Rawish *et al*.^1^ entitled ‘SGLT2 inhibitors are associated with improved long-term survival in Takotsubo syndrome: insights from large-scale real-world data’.^1^ In this trial emulation study based on real-world data from the TriNetX global network, the authors report an association between SGLT2 inhibitor (SGLT2i) use and reduced long-term mortality in patients with Takotsubo syndrome (TTS). While the authors already provide a thoughtful discussion of the potential biological mechanisms underlying this observation, we would like to further expand on the pathophysiological and translational evidence supporting this observed benefit.

Several mechanisms have been involved in the development of the characteristic acute regional myocardial contractile dysfunction observed in TTS, including excessive sympathetic stimulation, elevated circulating catecholamines, coronary microvascular dysfunction, multivessel epicardial spasm, and hormonal influences.^2^ Among these, the central role of catecholamines is supported by markedly elevated plasma levels in affected patients, as well as by the well-documented occurrence of TTS following pheochromocytoma crises or exogenous catecholamine administration.^2^ At supraphysiological concentrations, epinephrine exerts paradoxical negative inotropic effects mediated through β_2_-adrenergic receptor (β_2_-AR) signalling, involving a switch from Gs- to Gi-protein coupling.^2^ This signalling shift not only contributes to myocardial stunning but also promotes downstream inflammatory and endothelial responses.

In this context, activation of adrenergic pathways enhances cytoadhesion molecule expression in inflammatory and endothelial cells and disrupts the endothelial glycocalyx, thereby facilitating leukocyte diapedesis and sterile myocardial inflammation.^2^ Furthermore, β_2_-AR Gi signalling stimulates endothelial nitric oxide synthase (eNOS), leading to excessive nitric oxide production and nitrosative stress, which amplifies endothelial dysfunction, inflammatory infiltration, and myocardial contractile impairment.^2^

Consistent with these mechanistic insights, cardiac magnetic resonance imaging and histopathological studies have confirmed that myocardial inflammation is a hallmark of the acute phase of TTS.^2^ Importantly, the magnitude and persistence of the inflammatory response appear to carry significant prognostic implications. We previously demonstrated that the occurrence of systemic inflammatory response syndrome in TTS is associated with greater myocardial injury, more severe systolic dysfunction, and worse clinical outcomes, including increased in-hospital and long-term mortality.^3^ The persistence of inflammatory burden at hospital discharge was also demonstrated as an independent predictor of 1 year mortality.^4^ Notably, persistent inflammation beyond the acute phase is not uncommon and has been linked to incomplete ventricular recovery and adverse long-term prognosis.^5^

Emerging evidence suggests a mechanistic intersection between inflammation, oxidative stress, and SGLT2 expression within the cardiovascular system.^6–8^ SGLT2 expression has been identified in cardiomyocytes, particularly in regions characterized by macrophage infiltration and low-grade inflammation, where it may contribute to pro-oxidant signalling. Moreover, pro-inflammatory cytokines have been shown to upregulate SGLT2 expression in endothelial cells, thereby perpetuating oxidative stress, endothelial dysfunction, and vascular inflammation.^6^

In a rat model of isoprenaline-induced TTS, we observed pronounced myocardial oxidative stress, macrophage infiltration, pro-inflammatory and pro-fibrotic signalling, along with systemic microvascular endothelial dysfunction.^9,10^ Interestingly, treatment with empagliflozin attenuated these pathological alterations, reducing oxidative stress, inflammatory activation, and endothelial dysfunction. These findings provide experimental support for the anti-inflammatory, antioxidative, and vasculoprotective properties of SGLT2 inhibition in the TTS setting (*Figure 1*).

**Figure 1:**
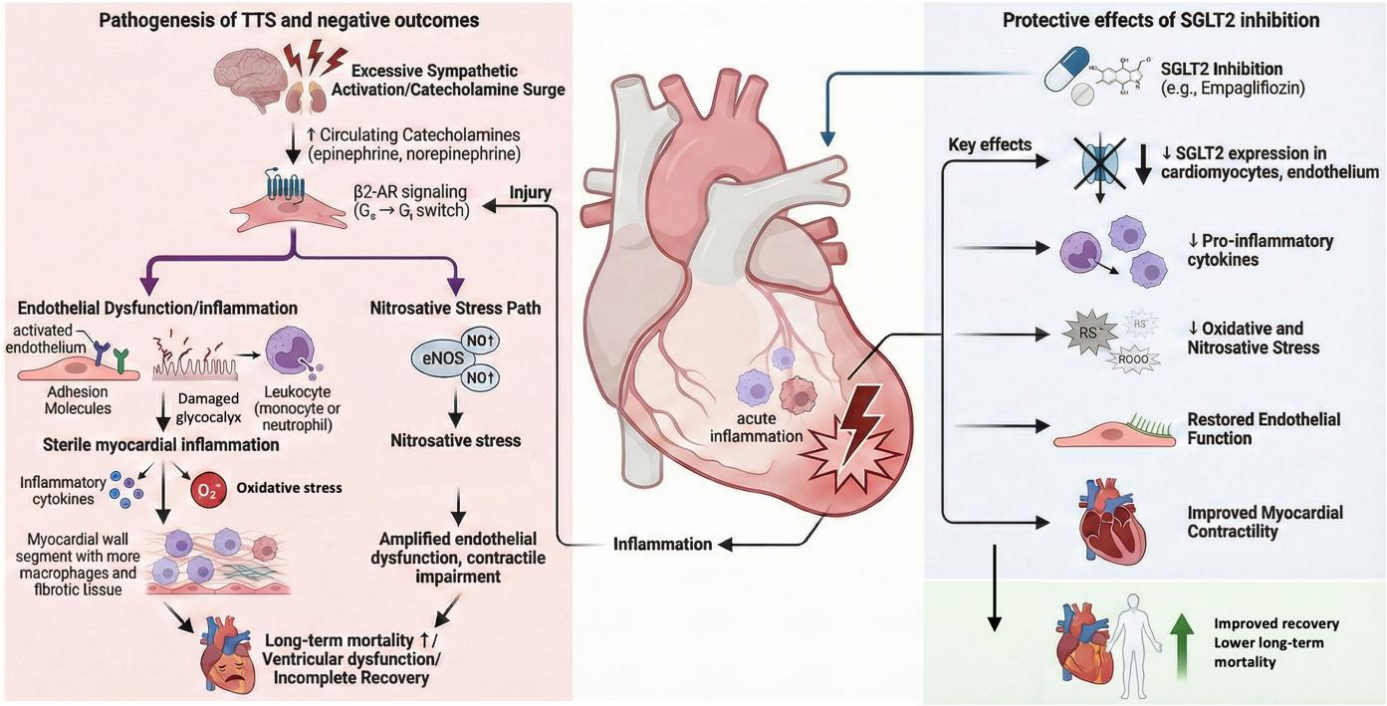
Proposed pathophysiological mechanisms in Takotsubo syndrome and potential protective effects of SGLT2 inhibition. Excessive sympathetic activation and catecholamine surge in Takotsubo syndrome lead to increased circulating epinephrine and norepinephrine, triggering β2-adrenergic receptor signalling alterations (Gs to Gi switch) and myocardial injury. These processes promote endothelial dysfunction, characterized by endothelial activation, glycocalyx disruption, and leukocyte recruitment, resulting in sterile myocardial inflammation and oxidative stress. Concurrently, activation of nitric oxide-related pathways contributes to nitrosative stress, further amplifying endothelial dysfunction and myocardial contractile impairment. The resulting inflammatory response within the myocardium may contribute to ventricular dysfunction, incomplete recovery, and increased long-term mortality. In contrast, sodium-glucose cotransporter 2 (SGLT2) inhibition may exert cardio protective effects through multiple mechanisms, including reduced SGLT2 expression and activity in cardiomyocytes and endothelial cells, attenuation of pro-inflammatory cytokine production, and reduction of oxidative and nitrosative stress. These effects may restore endothelial function, improve myocardial contractility, and ultimately promote improved recovery and lower long-term mortality in patients with Takotsubo syndrome.

Taken together, the convergence of mechanistic, preclinical, and large-scale observational data strengthens the biological plausibility of the survival benefit associated with SGLT2i reported by Rawish *et al*.^1^ These findings underscore the need for prospective randomized clinical trials to confirm causality, define optimal timing of initiation, and identify patient subgroups most likely to benefit from this therapeutic strategy.

## Data Availability

There are no new data associated with this article.
